# Supplementary material for: SGCD Missense Variant in a Lagotto Romagnolo Dog with Autosomal Recessively Inherited Limb-Girdle Muscular Dystrophy
Source: Genes (Basel). 2023 Aug 18;14(8):1641. doi: 10.3390/genes14081641 (PMC10454570; doi:10.3390/genes14081641)
Supplement: Supplementary file 1 [file genes-14-01641-s001.zip › table S1.pdf]

Table S1. The table shows the technical specifications of the primary antibodies used for immunohistochemistry.

Specifity	Clone	Company	Dilution	Antigen recovery
Dystrophin C-terminus	polyclonal	abcam	1:100	Heat 36 min at 95° C, Tris EDTA pH 9
Dystrophin Rod-domain	13H6	abcam	1:30	Heat 36 min at 95° C, Tris EDTA pH 9
Dystrophin N-terminus	34C5	Novocastra	1:20	Heat 36 min at 95° C, Tris EDTA pH 9
Alpha sarcoglycan	AD1/20A6	Novocastra	1:100	*
Beta sarcoglycan	βSarc/5B1	Novocastra	1:100	*
Delta sarcoglycan	polyclonal	abcam	1:100	Heat 36 min at 95° C, Tris EDTA pH 9
Gamma sarcoglycan	35DAG/21 B5	Novocastra	1:100	
Anti-Laminin	polyclonal	abcam	1:50	*
Spectrin	RBC2/3D5	Novocastra	1:100	*

*: used on frozen tissue
