# Supplementary figures and images for: SGCD Missense Variant in a Lagotto Romagnolo Dog with Autosomal Recessively Inherited Limb-Girdle Muscular Dystrophy
Source: Genes (Basel). 2023 Aug 18;14(8):1641. doi: 10.3390/genes14081641 (PMC10454570; doi:10.3390/genes14081641)

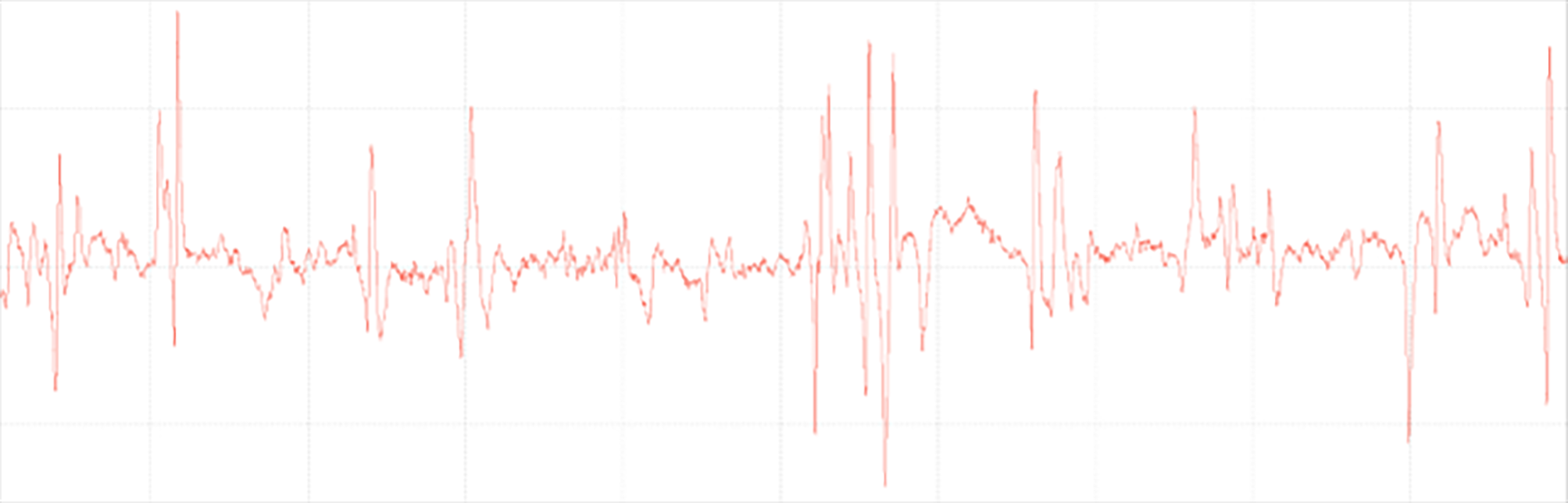

Supplement: Supplementary file 1 [file genes-14-01641-s001.zip › figura S1.tif]

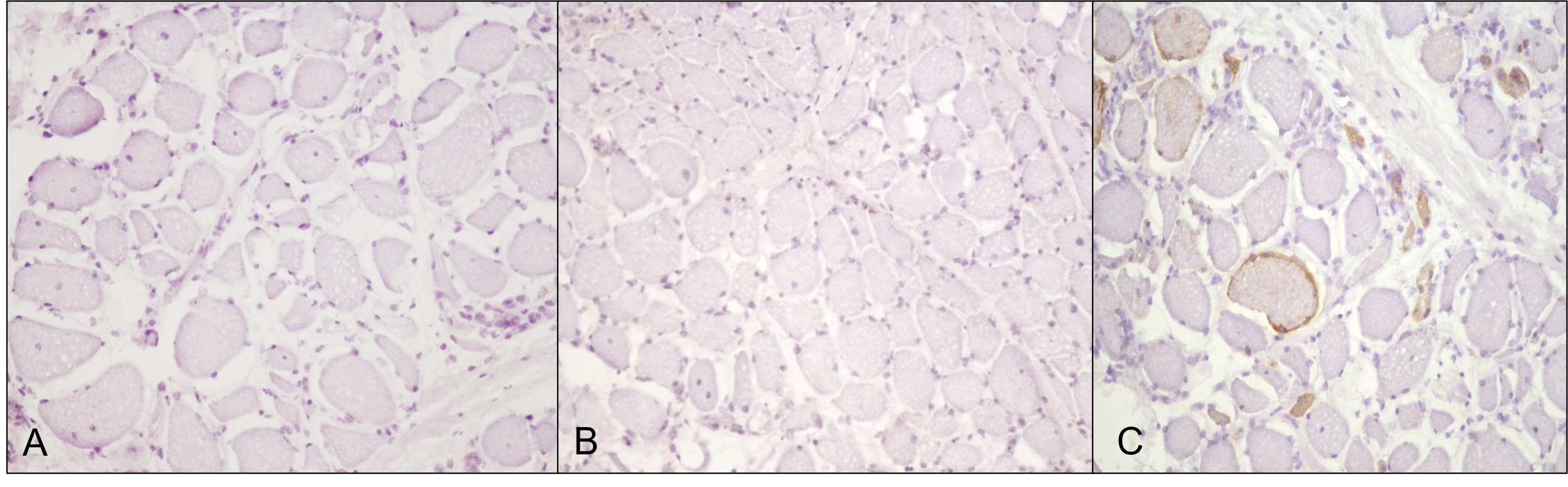

Supplement: Supplementary file 1 [file genes-14-01641-s001.zip › Figure S2.tif]
